# Recovering from the Ebola crisis: ‘Social Reconnection Groups’ in a rural Liberian community

**DOI:** 10.1017/gmh.2019.13

**Published:** 2019-08-13

**Authors:** M. Morelli, G. Cyrus, I. Weissbecker, J. Kpangbai, M. Mallow, A. Leichner, E. Ryan, R. Wener, J. Gao, J. Antigua, A. C. Levine, F. Feuchte

**Affiliations:** 1 California School of Professional Psychology, San Francisco, CA, USA; 2 International Medical Corps, Monrovia, Liberia; 3 International Medical Corps, Washington DC, USA; 4 Restore Hope, Monrovia, Liberia; 5 International Medical Corps, Amman, Jordan; 6 The University of Edinburgh, Edinburgh, UK; 7 Medical School of Brown University, Providence, RI, USA; 8 Deutsche Gesellschaft für Internationale Zusammenarbeit GmbH (GIZ), Monroiva, Liberia

**Keywords:** Collective trauma, Ebola virus disease, Liberia, mental health and psychosocial support, resilience

## Abstract

In 2014/2015, International Medical Corps (IMC) operated two Ebola Treatment Units (ETUs) in Liberia and three in Sierra Leone when the Ebola virus disease epidemic killed over 11,000 people across Liberia, Sierra Leone and Guinea. As Ebola cases declined in Liberia, IMC Psychosocial teams transitioned to working in communities highly affected by the epidemic. This article describes IMC's experience with developing and implementing a community-based mental health and psychosocial group intervention in a rural, severely affected Liberian town – Mawah – where 46 out of approximately 800 community members were infected, 39 of whom died. In this paper, we present how the group intervention, named ‘Social Reconnection Groups’, was developed and implemented. We then discuss intervention strengths, challenges, key lessons learnt and recommendations for how Social Reconnection Groups can be adapted for use in similar settings.

## Background

### EVD and the EVD response in Liberia

Ebola virus disease (EVD), an acute haemorrhagic fever in humans with an average case fatality rate of 50% (WHO, 2018), rapidly spread from the first recorded case in Guinea on 23 March 2014, to become the largest and most complex Ebola outbreak since the virus was first discovered in 1976. Over the course of the next 2 years, EVD would infect an estimated 28 616 people in Sierra Leone, Liberia and Guinea, with approximately 11 310 reported deaths (WHO, 2014–2015), although arriving at accurate estimates were hindered by difficulties in body tracing and tracing (McNamara *et al*. 2016; Cori *et al*. 2017).

The public health response to the outbreak was organized as five equally important and complementary pillars, including Beneficiary Communication and Social Mobilization; Contact Tracing and Surveillance; Psychosocial Support; Case Management; and Safe and Dignified Burials and Disinfection (WHO, 2014; Cooper, 2015; IFRC, 2015). At the national level, the Liberian Ministry of Health and Social Welfare led the psychosocial pillar, with multiple NGOS and agencies active in the mental health and psychosocial (MHPSS) response, both in terms of direct service provision and the training of local health workers (HC3, 2017).

Psychosocial activities that raised awareness about Ebola and reduced fear were high priorities. For example, in the outbreak's initial stages and throughout the epidemic, many refused to send sick loved ones to Ebola Treatment centres (ETUs) due to fears and myths about EVD, such as denials about the outbreak's existence, bad treatment in the ETUs and a fear of dying alone without informing family (Weissbecker *et al*. 2018). In some cases, community reactions and caregiving practices influenced the ability to contain infectious disease. Directives from the coordinated response to not touch the sick nor the deceased during burials also led to confusion and sometimes norms-violating behaviour, resulting in shame, guilt, anger and blame (Feuchte, 2015).

International Medical Corp (IMC) engaged in community outreach, education and psychosocial support to EVD patients admitted to the ETUs and their families and follow-up support for re-integration of survivors. Health staff working at ETUs received basic support such as stress management informational sessions and individual support as needed (Weissbecker *et al.*, 2018). Other community outreach workers also trained in supportive communication and psychological first aid conducted door-to-door visits of suspected Ebola cases and engaged in dialogue with community leaders to addresses stigma, rumours and cultural misperceptions about the disease. Solidarity kits were also given to Ebola contacts, survivors and their families who lost material goods from disinfection and were suffering from social exclusion and stigma (IFRC, 2015).

While these and other activities made a positive impact in alleviating psychosocial-related stressors, survivors and others were often left coping with profound loss, fear, mistrust and conflict in their families and communities. Research suggests that the simultaneous experience of such intense fear, powerlessness, loss of control and threat of death can overwhelm coping abilities, disrupt belief systems and fragment memories in individuals (Herman, 1992; van der Kolk, 2015). In addition, traumatic events experienced by groups of any size, including whole communities, have been shown to lead to widespread distrust, loss of motivation, and disconnection from cultural identity, which can then be passed on to subsequent generations (Yellow Horse Brave Heart, 2003; Whitbeck *et al*. 2004; Leary, 2005; Volkan, 2006; Somasundaram, 2014).

Therefore, as agencies learned more about how individuals, families and communities were impacted, psychosocial teams sought to explore approaches that would support communities in their long-term recovery. To do this, it would be important to not only address vulnerabilities that can result from traumatic stress, but to also anchor recovery efforts in the resilience and strength that communities showed. Accounts and case studies show how local communities quickly adapted, often in the face of extreme stressors and the absence of a well-supported healthcare infrastructure or sophisticated containment techniques, to create innovative solutions that helped contain the spread of infection at a local level (Abramowitz *et al*. 2015; Kutalek *et al*. 2015).

The following article is a case study, which aims to describe IMC's experience in developing and implementing a community-based psychosocial intervention in response to the MHPSS needs of the severely affected Liberian town of Mawah, where 46 out of approximately 800 community members were infected, 39 of whom died. Key lessons learnt and limitations, challenges and risks to this approach are also discussed in order to inform the MHPSS work of future responses to epidemics.

### The Town of Mawah

Mawah, a rural town with a population of about 800, resides in the Fuamah District of Bong County, approximately 130 km driving distance to the capital Monrovia. Mawah is surrounded by fertile forest and farms and bordered by the Guinea River and branching creeks. Most families' incomes fall under the poverty line, with income coming mainly from palm oil, palm wine, crawfish, cold water fish and crab.

In August 2014, the outbreak reached Mawah, where over a span of few weeks, 46 community members were infected – 39 of whom died. When the EVD task force, directed by the County Ministry of Health and Social Welfare office, was notified of the outbreak, a 21-day community quarantine led to restricted transit and closure of markets (Nyenshwah *et al*. 2015).

IMC first learned of Mawah while treating some community members at the Bong County ETU, Suakoko District (170 km driving distance) and communicating with local authorities to take measures to support food security and prevent infection. In December 2014, IMC invited residents from Mawah and other impacted communities to a workshop exploring current needs and available options to address them. It was during this workshop that the participants from Mawah raised the possibility of a counselling intervention in their community.

In response, IMC Psychosocial Staff members travelled to Mawah in January 2015 accompanied by a psychosocial counsellor from Bong Mines Hospital who was well known and respected in the community. The team first met with community stakeholders, included the town chief, the women's organization, elders and others. The town elders then chose the participants to take part in the initial focus group discussions, before the intervention was designed and implemented. At these focus group discussions, which were an open forum for community members to express their needs in response to the epidemic, it was decided to hold counselling session with support from IMC.

To further assess the psychosocial needs in the community, IMC staff used a questionnaire and conducted semi-structured interviews with town elders, heads of community-based organizations and community members about how EVD epidemic affected community members and what community life was like and what structures existed before the epidemic.

Initial interviews and discussions showed that people in Mawah are proud of their music, creativity, strong work ethic, talented dance-troupes, skilled soccer players and strength of societal organizations. The women's organization – which primarily takes disciplinary actions, resolves conflict and engages with local NGOs to address community needs – is an integral part of a complex power structure in Mawah.

Community members described how, before the outbreak, they would visit and check up on each other's welfare after returning home from the farms; in times of hardship, the community would come together to problem solve and pool resources. Mawah's sense of community is a long tradition. For example, Mawah, a Gola word for ‘have you decided to come back?’, represents the reunification of the town's founding brothers after one brother's unsuccessful attempt to establish himself on one side of the river. Current residents described how something that affects one person affects them all.

Mawah residents reported several and interrelated outbreak-related stressors, such as feelings of fear, panic, lack of community-based support, loss of breadwinners, economic and food insecurity, increased conflict and disrupted social cohesion. Residents described guilt, shame and helplessness from watching loved ones die.

During these initial interviews, residents also reported overwhelming grief, ‘disrupted togetherness’, unresolved conflict, inability to work and a breakdown of Mawah's traditional way of resolving disputes. Many members of Mawah described Ebola using words translated from Kpelle, the local language, as ‘hatred’, ‘kill all’ and ‘separation’. Residents told how their previously united community was overwhelmed by the sense that doomsday had arrived. Citing ‘love’ as the reason, community members resolved to not flee and remain to take care of each other – for example, by sharing in caretaking duties and pooling money to visit family members at ETUs.

During the early days of the epidemic, particularly when facilities were overwhelmed, some families reported that they only learned about the death of a loved one in ETUs through persistent follow-up. They described how the lack of communication and culturally dishonourable treatment of the deceased (usually cremation) at some ETUs fuelled public distrust and created resistance to cooperation in other communities. Official messaging to protect from Ebola infection included directives to not hug and touch each other and keep distance from those who are sick or buried, which is the opposite of what people in Mawah would traditionally do. Community members described how such changed behaviour often caused anger and distress.

Market closures, limited movement and halted farming activities led to spoiled excess crops and a scarcity of food. People in Mawah described how vendors stopped coming in to exchange goods due to fear, stigma and quarantine measures. Community members withdrew from each other, hugging and touching stopped and people sat far away from each other in church and mosque. Schools closed, restrictions were placed on public gathering places and games and activities normally played with the members of neighbouring towns stopped. (In 2015, a documentary film about the epidemic was created, as well as an accompanying interactive website with more stories about the impact on Mawah (Pulitzer Center, 2015). Find more information, as well as a link to the website, here: https://pulitzercenter.org/reporting/mawah-when-ebola-came-toour-village.)

### Development of social reconnection groups

IMC sought to support community members who had problems returning to their day-to-day functioning by providing a safe platform that participants could use to reflect on their experiences, build trust, address resulting problems and move towards re-establishing mechanisms of social support which existed before the outbreak. The approach aimed to also make sense of difficult and distressing memories, mourn lost loved ones, come to a shared narrative of their collective experience and shift towards a future with hope.

Our community-based approach, later called ‘Social Reconnection Groups’, developed from Sociotherapy, which is a group model that focuses on daily stressors, problem solving, resolving conflicts between family and community members, effective ways of coping and generating mutual emotional support (Richters *et al*. 2013; Verduin *et al*. 2014; IASC, 2015). With origins from Therapeutic Community as Method, also described as ‘community acting as a doctor’ (Rapoport, 1960), Sociotherapy principles that guide practical guidelines include two-way communication at all levels, decision-making at all levels, shared leadership, consensus in decision-making and social learning by social interaction in the here-and-now (Richters *et al*. 2008).

Sociotherapy was chosen as an underlying model also because of its flexibility to integrate various activities from different theoretical perspectives. EVD awareness and education fostered factual information about EVD prevention and transmission. Psychoeducation was also provided to normalize the diversity of mourning reactions. Local customs and traditions were respected and re-established where possible, such as Town Hall, which is a large, covered meeting place where community members congregate and community-level discussions, announcements, decisions, music and events take place.

Community Healing Dialogues (CHDs) were a precursor to Social Reconnection Groups. Also drawing on sociotherapy as an underlying model, they focused on individual psychosocial wellbeing, as well as strengthening community relationships and community capacity to handle problems (WHO, 2017). CHDs were initially introduced in Liberia in September 2014 with support from the Urgent Action Fund when six Mental Health Clinicians were trained by Dr Florence Baingana to provide such dialogues. From October 2014, the Ministry of Health with the support of WHO scaled up the approach to the Ebola-affected counties, with the Carter Center also introducing CHDs that included Town Halls. These community-based interventions were included as key recommendations to support communities in the post-emergency phase (IASC, 2015).

Inputs were also taken from a group intervention manual from the Center for Victims of Torture that was used after the wars in Liberia, as some of the IMC group facilitators had been trained in this approach (Barry & Pearson, 2004). Research on community interventions after wars and genocide summarized key ingredients for successful recovery support in the RICH framework: Respect, Information, Connection and Hope (Pearlman, 2013). By the end of the intervention, Sociotherapy had been complemented with EVD awareness and education, psycho-education, peace and reconciliation, livelihood building, and strengths and resilience-based activities.

### Facilitators and training

The IMC Program Director with Doctoral degree in Psychology (last author) and IMC Psychosocial Coordinators with degrees in Psychology or Social Work (first and seventh author) provided training and supervision prior to implementation, including several meetings, a 1-day workshop and then ongoing. Programme staff and group facilitators met weekly to discuss the previous week's groups, process clinical encounters and plan for ensuing sessions. IMC Liberian national staff (second and fourth author and others) significantly contributed to the programme due to their knowledge of local customs and context. Facilitators were Liberians with degrees or experience in social work, psychiatric nursing or mental health, with extensive experience working in conflict and post-conflicts settings.

Salient aspects of training include Sociotherapy principles and adaptation to an EVD context in Liberia; group facilitation and fostering safety and cohesion; resilience, strengths-based and recovery-oriented approaches; counselling and communication skills; stress management, self-care and noticing one's limits; risks, conflict mediation and anger management; dealing with grief; and coping skills. Incorporated training topics were consistent with national standards (IASC, 2015).

Either the IMC MHPSS Program Director or Coordinator was present at all sessions to offer support. Initially, group facilitators consisted of three IMC Psychosocial staff and the Psychosocial Counsellor from nearby Bong Mines Hospital. A second facilitator per group was later recruited from the IMC psychosocial team and county-based social workers. Additional facilitators received individualized training by the IMC Program Director. Group facilitators kept records of each session, which was compiled and reviewed by IMC Program Director and Psychosocial Coordinators, and kept at the IMC Bong County ETU.

### Group selection

Group selection of the four small groups each with 14–17 participants was self-selected and/or randomly selected. A total of 60 residents participated in the small groups, with no attrition. All group participants were able to attend all groups, requesting to skip one Saturday due to voting in local elections. It is unknown how many residents were not able to participate due to the necessity to work and acquire basic needs. Also, records were not kept of those who participated in Town Hall and not the groups. While it is also unclear how many beneficiaries this project indirectly reached, the community of a population of 800 all reside within close proximity to each other and comprise a tight-knit social network.

Informed consent was received from all group participants, who were given detailed information about the purpose, content and possible risks of the activity before they agreed to take part. Each group had a mix of sexes, ages, religion and background/social status. The local Imam and Pastor both took part in the same group. The main language was Kpelle, with some groups and the larger forum also using Liberian English in parallel, with people translating between the languages.

### The sessions

Sessions were held on Saturdays over 10 weeks, from late January to early April 2015. The day began at approximately 09:30 in Town Hall, where participants, staff and community members would congregate. Town Hall would start with traditional music, dancing, singing and prayers led by both Imam and Pastor. Introductions of new visitors, announcements and ongoing EVD education and awareness also took place in Town Hall. At 10:30, the four small groups would break out and meet in separate locations. At 12:30, the small groups would re-join the large group in Town Hall. Announcements, community-led discussions and decisions, traditional music, prayers and a shared lunch would mark the day's end.

Whereas the large group in Town Hall focused on issues that would impact the large group and community, the smaller group sessions gave participants a space to talk about whatever they chose. The *first small group session* focused on building trust, safety and cohesion, through reaching consensus on group ground rules, emphasizing that sharing of personal experiences is not obligatory, ensuring participants understand intervention objectives, and asking participants about their expectations for how they hope participating in the intervention would help.

The *next two sessions*’ participants shared in-depth how they, their families and the community dealt with the outbreak and what changed because of Ebola. Many discussed how the community unknowingly took in the first person infected with Ebola, thinking the illness could be treated by Mawah's traditional healer. As people shared their personal stories, group participants responded with empathy and encouraging words and gestures. Many also discussed having nightmares and avoiding places that reminded them of the deceased, such as the youth avoiding the soccer field. When flashbacks, nightmares and various grief reactions came up, facilitators attempted to foster containment and an emotionally safe space and talked about the diversity of grief symptoms to normalize these reactions as much as possible.

EVD misinformation, cultural misperceptions of the disease and directives from the coordinated response to not touch the sick nor the deceased during burials led to confusion, norms-violating behaviour and feelings of shame, guilt, anger and blame, which sometimes led to conflict. *Sessions 4 and 5* included topics of anger management, strategies to resolve conflict and identifying community conflict resolution structures that exist or can be created in the community.

*Session 6* focused on strengths, values and coping – at the individual, family and community levels. These groups identified coping mechanisms, reflected on how values govern decision-making processes and recognized how strengths are used to cope with difficult experiences. Livelihood building discussions led to action during discussions of Mawah's strong work ethic, and their collective response by not fleeing at the peak of the outbreak.

*Sessions 7, 8 and 9* focused on looking towards the future, while also reflecting on what had happened so far. In these sessions, facilitateddiscussions focused on how conflicts would be addressed in the community after IMC leaves. Group participants discussed what topics remained to be addressed, reflected on stated objectives from the first session, summarizing what has been learned and discussing whether they have been achieved.

During these sessions, each small group also selected two facilitators among themselves to lead the remainder of the process. They discussed how to continue after completion of the Social Reconnection Groups, and three small groups decided on continuing to meet and to collaborate in the cultivation of peanut or pepper. The fourth group decided on strengthening the community-based conflict resolution mechanisms. Group participants also discussed how they wanted to celebrate the closing session.

The final small group session, *session 10*, was facilitated by community members. Participants gave feedback on groups, addressed pending issues and made plans to continue group participation. In the large Town Hall meeting, group participants role-played key lessons and highlights. Traditional dance and music marked the closing.

### Observations of the process

Facilitators noted that early sessions of the groups were extremely tense and filled with tears, as group members told painful stories, clarified misinformation and confusion, and addressed interpersonal conflict. This initial intensity led to the decision to add a co-facilitator to each group, support positive coping as early as possible and provide structure by including pre-determined activities. IMC nurses and doctor visited some group sessions, provided feedback to medical-related questions that group participants had about Ebola, and validated the strength of Mawah's response in the face of such a deadly disease.

When sessions were dominated by some participants, facilitators asked others to speak if they felt comfortable doing so, while consistently reiterated that talking was not obligatory. Many men struggled with showing emotion. Participants, as well as staff, compared Ebola with the Liberian Civil War, suggesting EVD presents different kinds of dangers. For example, a staff member expressed that ‘at least in the war you know where the enemy is and where to run from. At least in the war you can comfort the scared, crying, and huddled up children, and touch your loved ones as they die and you say goodbye.’

Many participants comforted, consoled and encouraged each other. After a few sessions, staff observed that the atmosphere in sessions and Town Hall forums became more lively, energetic and relaxed as compared to previous weeks. In some cases, participants would run to prepare session rooms before staff arrived and start the sessions themselves. In the Town Hall forums, staff observed more smiling, dancing, laughing and sharing jokes.

Group participants expressed frustration and anger when NGOs distributed money and supplies to survivors and families. Community members were offended, expressing that all community members were impacted by EVD. (For example, community members would pool together all money needed to support transportation to ETUs.) Some participants were also disappointed to not receive ‘sitting fees’ for participation in the Social Reconnection Groups, which many expected from participating in the groups. IMC did not take this approach in order to reinforce self-motivation and to let participants attend the groups for intrinsic value.

After the town hall meetings, all SRG facilitators and the supervisors met briefly to reflect on the experiences of the day, check for any need of support and collect key points for the upcoming training session. One Mawah resident expressed in the final feedback that he did not like these ‘secret meetings’ of facilitators. Other critical points included not receiving financial compensation for having attended the Social Reconnection Groups.

Towards the seventh and eighth sessions, participants initiated collaboration on livelihood projects, such as peanut farming or pepper farming. Groups began working the farms to clear debris. The youth ‘paid tribute to the youth that fell’ by organizing a football match. A Peace Committee was set up to help resolve and settle disputes. New group facilitators effectively assumed responsibilities and facilitated open discussion groups in the final weeks, with topics that focused on moving forward after IMC would leave, and how to work together on livelihood practices. Three of the groups continued after intervention closure. Examples of positive and critical comments gathered as part of routine programme monitoring and evaluation activities are included in Box 1.

Box 1. Quotes about the ‘Social Reconnection Groups’ post-intervention

Positive Examples

‘The Ebola brought hatred among us, but the workshop has put us together. We have learned how to counsel other people.’

‘It made us to overcome the grief of Ebola and go back to normal activities’

‘I learned that with grudge, we cannot progress and we also learned to prevent Ebola as well as equality and sharing; we learned to control our anger and forgive others.’

‘We have learned how to talk to each other and be friendly with one another. We also learned coping strategies such as breathing slowly.’

‘Right now I am talking to my wife politely and even my children but before I used to give them tough time. I'm feeling well, because when I think of doing something different, but when I think about the group I come to myself.’

Critical Examples

‘Even though they told us that they will not give us anything, but we were expecting sitting fees, which were not given.’

‘The only thing that hurt me is that IMC did not give us anything at the end of the ten weeks.’

‘They did everything including bringing food for lunch, but for the length of time we were there, we did not receive any little cash for soap. We felt bad.’

‘I don't like the aspect of the workshop where the facilitators used to leave us and go to secret meetings. It means that they used to go to discuss and deprive us of our end of workshop benefit.’

Box 2. Possibly important ingredients of the Social Reconnection Groups

- Adaptability to integrate culturally responsive methods, such as Town Hall discussions and communal music and movement
- Reflecting the resilience of individuals, families and wider community
- Delivering accurate information and clarifying confusion about Ebola
- Conveying empathy and normalizing reactions to extreme stress
- Facilitating acceptance and mourning loss
- Fostering cohesion and facilitating hope and interpersonal learning
- Restoring livelihood practices

### Strengths of the social reconnection groups

Strengths of the Social Reconnection Groups include the flexibility to integrate activities specific to local custom and context. Town Hall forums, in particular, provided a platform to bring community members together and help them come to an empowering collective narrative, such as how community members resolved to take care of each other and pool resources during the peak of EVD epidemic. Town Hall meetings also allowed for communal music, rhythm and movement, which can all have important therapeutic value (Van der Kolk, 2015).

The ability to make meaning of events and weave them into a coherent life narrative has shown to help people cope with traumatic events (Herman, 1992). Finding new meaning helped balance the detrimental aspects of EVD with some of the positive, such as people volunteering, caring and showing that Liberia is not alone. A major strength was also the commitment and skill of group facilitators, who were also facing loss, isolation and ongoing stigma from their home communities. Staff, particularly those that spoke the local language, helped build rapport with the community and improve international staff's understanding of local culture and practices.

### Limitations, challenges and risks

The main limitation of this programme includes the inability to assess for and refer persons with mental disorders or address all individual issues. Some group participants stayed relatively quiet and/or did not want to talk about their experiences. While efforts were made not to probe participants, the number of participants who felt obligated to share their experiences remains undetermined. When individual counselling services were requested and efforts were made to liaise with other organizations that offered these services, it was unclear if those services were rendered, and what the effects were.

Additionally, it is unclear how pre-existing power structures were disrupted, or how inequalities were reproduced. Mawah specifically, and Liberian communities in general, have complex power structures. For example, the Town Chief died of EVD, potentially creating political instability during the time that IMC intervened. While IMC tried to anticipate and address false expectations, many group participants expressed disappointment at not receiving ‘sitting fees’.

Community members who were not selected to participate in the groups expressed disappointment. While all community members were free to take part in the group sessions, we do not know what internal community dynamics may have prevented or excluded some people from attending. In the future, it may be helpful to more closely work with different community groups and find ways to ensure social inclusion.

Finally, the dynamics between men and women were not systematically considered. In the beginning, it was discussed to have separate groups for men and women, but the women especially insisted that they prefer mixed groups. At one point, a female facilitator reflected on her difficulty managing verbal and non-verbal dominance of a man in her group and was additionally unsure how to react to a men's tearfulness.

### Key lessons learnt

Key lessons include the need for improved mapping, coordination and follow-up of referral services rendered, as well as more thorough monitoring and evaluation strategy to help us better understand short- and long-term effects of the group intervention. Elucidating mediators of change – for example, how re-establishing specific cultural and traditional practices that already existed like the Town Hall forum can contribute to coping with psychological distress and the resolution of trauma and conflict– could provide crucial information when designing approaches in the future.

Lastly, a more thorough and transparent process of identifying group members and clarifying incentives would minimize confusion and potential resentment as the intervention progresses. Also, a follow-up analysis of how Mawah has recovered post-intervention would be invaluable to understand what were the most and least helpful aspects of the Social Reconnection Groups.

While more work needs to be done to clarify some of the results of the intervention, we see many strengths in this approach and would recommend this flexible and adaptable community-based approach for use in similar settings. We also recommend collaboration with government officials, particularly if this approach is scaled up, as their support and involvement were very important in the planning and implementation stages of this programme. For example, the Psychosocial Counsellor from nearby Bong Mines Hospital, a respected and trusted member of the local community, was instrumental in gaining the trust of Mawah and other local community members during the initial assessment and planning stages. This type of collaboration is needed at many levels, especially as we look to understand and implement effective approaches to social reconnection.
